# How do taxi drivers expose to fine particulate matter (PM_2.5_) in a Chinese megacity: a rapid assessment incorporating with satellite-derived information and urban mobility data

**DOI:** 10.1186/s12942-024-00368-5

**Published:** 2024-04-13

**Authors:** Shuangming Zhao, Yuchen Fan, Pengxiang Zhao, Ali Mansourian, Hung Chak Ho

**Affiliations:** 1 School of Remote Sensing and Information Engineering, Wuhan University, Wuhan, China; 2 GIS Centre, Department of Physical Geography and Ecosystem Science, Lund University, Lund, Sweden; 3 Department of Public and International Affairs, City University of Hong Kong, Hong Kong, China

**Keywords:** Taxi drivers, PM_2.5_ exposure, Spatiotemporal analysis, Satellite-derived information, Urban mobility data

## Abstract

**Background:**

Taxi drivers in a Chinese megacity are frequently exposed to traffic-related particulate matter (PM_2.5_) due to their job nature, busy road traffic, and urban density. A robust method to quantify dynamic population exposure to PM_2.5_ among taxi drivers is important for occupational risk prevention, however, it is limited by data availability.

**Methods:**

This study proposed a rapid assessment of dynamic exposure to PM_2.5_ among drivers based on satellite-derived information, air quality data from monitoring stations, and GPS-based taxi trajectory data. An empirical study was conducted in Wuhan, China, to examine spatial and temporal variability of dynamic exposure and compare whether drivers’ exposure exceeded the World Health Organization (WHO) and China air quality guideline thresholds. Kernel density estimation was conducted to further explore the relationship between dynamic exposure and taxi drivers’ activities.

**Results:**

The taxi drivers’ weekday and weekend 24-h PM_2.5_ exposure was 83.60 μg/m^3^ and 55.62 μg/m^3^ respectively, 3.4 and 2.2 times than the WHO’s recommended level of 25 µg/m^3^. Specifically, drivers with high PM_2.5_ exposure had a higher average trip distance and smaller activity areas. Although major transportation interchanges/terminals were the common activity hotspots for both taxi drivers with high and low exposure, activity hotspots of drivers with high exposure were mainly located in busy riverside commercial areas within historic and central districts bounded by the “Inner Ring Road”, while hotspots of drivers with low exposure were new commercial areas in the extended urbanized area bounded by the “Third Ring Road”.

**Conclusion:**

These findings emphasized the need for air quality management and community planning to mitigate the potential health risks of taxi drivers.

## Introduction

Population exposure to air pollution is a concept regarding how the local population can suffer from ambient pollution exposure during a particular length of time causing unfavorable health conditions [2, 6, 21, 36]. Based on the definition of the United Nations Office for Disaster Risk Reduction (UNDRR), exposure is the “situation of people, infrastructure, housing, production capacities and other tangible human assets located in hazard-prone areas”, which implies that population exposure involves environmental quality as well as population distribution and mobility, especially in high-density areas with high-density living, such as China. Specifically, China has experienced high pollution levels due to urbanization and industrial development [14]. Even on a low air pollution day, the pollution level exceeds WHO’s air quality thresholds or China air quality guidelines, inducing severe health risks across cities in China [5, 37]. Thus, previous studies have attempted to combine population datasets and urban mobility data (e.g., mobile phone data, bike trajectories) to estimate exposure to air pollution among various populations (e.g., general population, bike riders) in areas with high-density living [1, 3, 10, 19, 31].

PM_2.5_ (particulate matter with a diameter < 2.5 µm) is one of the riskiest air pollutants worldwide [9, 13, 27], especially in China [11]. Specifically, PM_2.5_ is traffic-related air pollution that results in long-term adverse effects on population health. Previous studies have modelled traffic-related PM_2.5_ pollution and related health impacts [16, 29, 31, 32]. For example, Tang et al. [32] incorporated traffic behaviors with land use regression to estimate dynamic air pollution exposure in Hong Kong, showing that increased mobility led to a 13% and 3% higher population exposure level to PM_2.5_ among working adults compared to the older adults and individuals aged < 18. These results indicated that robustly characterizing the dynamic population exposure among vulnerable subpopulations is important for health management and urban planning.

Among all vulnerable individuals, taxi drivers are one of the most understudied groups despite their high exposure risk due to their job nature. Some studies have applied a panel design with a small number of participants to quantify exposure levels among taxi drivers in Europe and the United States [8, 44]. For example, Zagury et al. [44] recruited 29 drivers in Paris to study their exposure level, reporting higher exposure level in taxis compared to the ambient air monitoring network and fixed stations nearby automobile traffic. Gany et al. [8] conducted a study with a hundred drivers in New York and found that the concentration of fine particulate matter (PM_2.5_) in taxis was higher than those in nearby central monitoring stations. Nonetheless, these studies were limited by data availability as air monitoring networks are usually sparsely distributed and cannot demonstrate the moving vehicle’s dynamic exposure (e.g., taxi). Moreover, the estimation of population exposure was restricted by the number of samples and participants as well as the techniques to record air quality in a taxi. More importantly, the population exposure level of taxi drivers could vary over time [23].

Therefore, this study developed a new method to rapidly assess the population exposure of taxi drivers based on open datasets of air pollution and big data from urban mobility information. Specifically, this rapid assessment combined information from representative stations of the air monitoring network, satellite-derived information from land use regression, and urban mobility data from taxi trajectories to estimate drivers’ PM_2.5_ exposure in Wuhan, China. The research objectives were: (1) to estimate hourly PM_2.5_ exposure of taxi drivers, (2) to compare whether representative monitoring stations may overestimate or underestimate the dynamic PM_2.5_ exposure of taxi drivers, and (3) to evaluate whether hourly exposure of taxi drivers in different scenarios (weekday, weekend) exceeded WHO’s and China (PRC)’s national guideline thresholds.

## Data and methods

### Study area

Wuhan, China was selected as our study area. This megacity has a high-rise, high-density built environment in central China covering ~ 8494 km^2^ and a population of over 11 million people. Wuhan is also a major transportation hub and a key gateway to other parts of China. There are three railway stations in Wuhan, Wuchang Railway Station, Hankou Railway Station, and Wuhan Railway Station which Wuchang Railway Station provides train services to all provincial capitals in mainland China and is the largest general-speed railway terminal in central China. Hankou Railway Station is responsible for the passenger transportation business of east–west trains originating and passing through the Wuhan hub. The three railway stations are all within the “Third Ring Road”, a 91-km long ring expressway connecting the passenger and freight hubs and forming the extended urbanized areas in Wuhan. The high-rise, high-density environment as well as transportation patterns are key factors influencing PM_2.5_ in Chinese cities [30, 41].

**Fig. 1:**
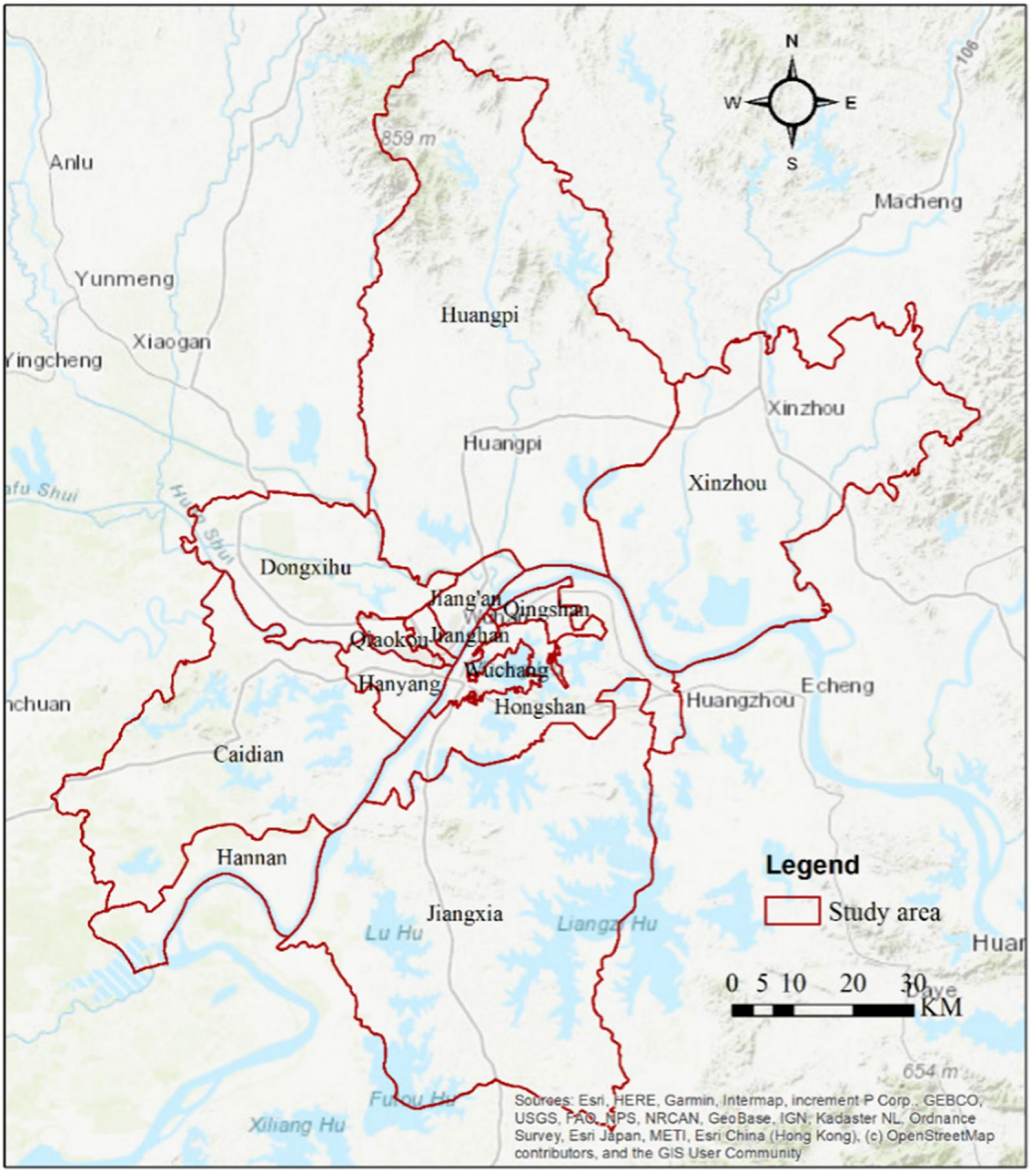
Study area—Wuhan, China. Wuhan comprises 13 districts (Wuchang, Hongshan, Jianghan, Jiang’an, Hanyang, Qiaokou, Qingshang, Jiangxia, Xinzhou, Huangpi, Dongxihu, Caidian, and Hannan) and the three main districts are Wuchang, Hankou, and Hanyang

Wuhan is also an economic, industrial, cultural, and educational center comprising 13 districts and the three main districts are Wuchang, Hankou, and Hanyang. Wuchang is the cultural and educational center of Wuhan, home to several universities and museums, whereas Hankou is the commercial center known for its bustling streets and vibrant nightlife. Hanyang is an industrial zone with many factories and manufacturing facilities (Fig. 1). These main districts are connected by the “Inner Ring Road”, a 28-km long ring road forming the most urbanized areas in Wuhan, which aims to provide rapid passenger transportation, thus increasing PM_2.5_ emissions in the city.

Consequently, Wuhan has notable air pollution and related health impacts. For example, a previous study investigated hourly air pollutants including PM_2.5_ in Wuhan between 2013 and 2014, suggesting that the average PM_2.5_ concentration needed to be reduced by at least 5% annually to achieve clean air quality by the end of 2017 [39]. A local study reported that a 10 μg/m increase in daily PM_2.5_ was associated with a 0.87% increase in cardiovascular hospital admissions between 2013 and 2015 [38]. Furthermore, another local study investigated hospital admissions between 2016 and 2018 and found higher health impacts from daily PM_2.5_ than the above study [29]. Specifically, this study found that a 10 μg/m3 increase in PM_2.5_ was associated with 1.23% and 1.95% increase in hospital admissions for cardiovascular and respiratory diseases in Wuhan. These results suggested that a study of PM_2.5_ exposure among taxi drivers in Wuhan is essential as the local population has a high risk of air pollution-related health issues, with traffic-related air pollutants being one of the major sources in Wuhan. Furthermore, the urban settings of Wuhan are similar to other megacities in China, thus this rapid assessment method could be applied in other locations.

### GPS-based taxi trajectory data

GPS-based taxi trajectory data was obtained from a taxi company in Wuhan for academic purposes. The data was collected from > 8300 taxis between May 13, 2014 and May 31, 2014, a non-pandemic period with air pollution extremes in Wuhan, which helps justify the adaptability of our proposed rapid assessment to other Chinese megacities with similar urban settings.

**Fig. 2:**
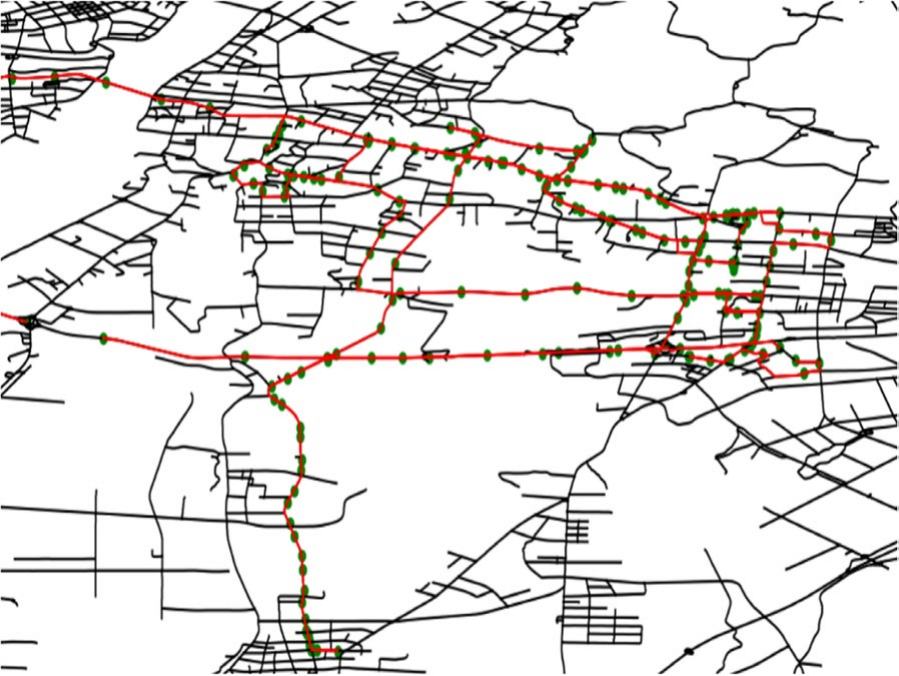
Map matching results based on the trajectory points of representative trips. Black and red lines represent the road network and the trajectories after map matching, respectively. Green points represent raw taxi trajectory points

Each taxi’s GPS recorded several trajectory variables approximately every 10 s or 1 min including taxi ID, sampling timestamp, the geolocation in longitude and latitude, heading direction, and passenger status (e.g., empty or occupied) [45]. Due to potential data bias and noise (e.g., abnormal GPS device, signal loss), raw taxi trajectory data were preprocessed to eliminate outliers, including data cleaning, trip extraction, and map matching. Trip extraction was conducted to recognize taxi trips based on passenger status information (occupied versus vacant). Map matching refers to the process of matching GPS trajectory points to a specific road network [4] to locate the above-mentioned GPS trajectory points of the target vehicle more accurately on the corresponding road segments, and to accurately obtain the vehicle’s movement trajectory. This study employed a map-matching method based on the Hidden Markov Model (HMM) [26]. Figure 2 presents the map-matching results based on the trajectory points of representative trips.

### Spatiotemporal variability of PM_2.5_ exposure

A rapid assessment framework based on a previous study [43] was proposed to reproduce spatiotemporal data of PM_2.5_ pollution to estimate dynamic exposure among taxi drivers. Specifically, this framework assumed that PM_2.5_ data from an annual map had high spatial variability but no temporal variations, whereas PM_2.5_ information from monitoring stations could have high temporal variability but not able to represent spatial variations due to a single location for each station. Thus, a combination of data from the annual PM_2.5_ map and information from representative monitoring stations may be able to rapidly reproduce spatiotemporal data for further assessment.

**Fig. 3:**
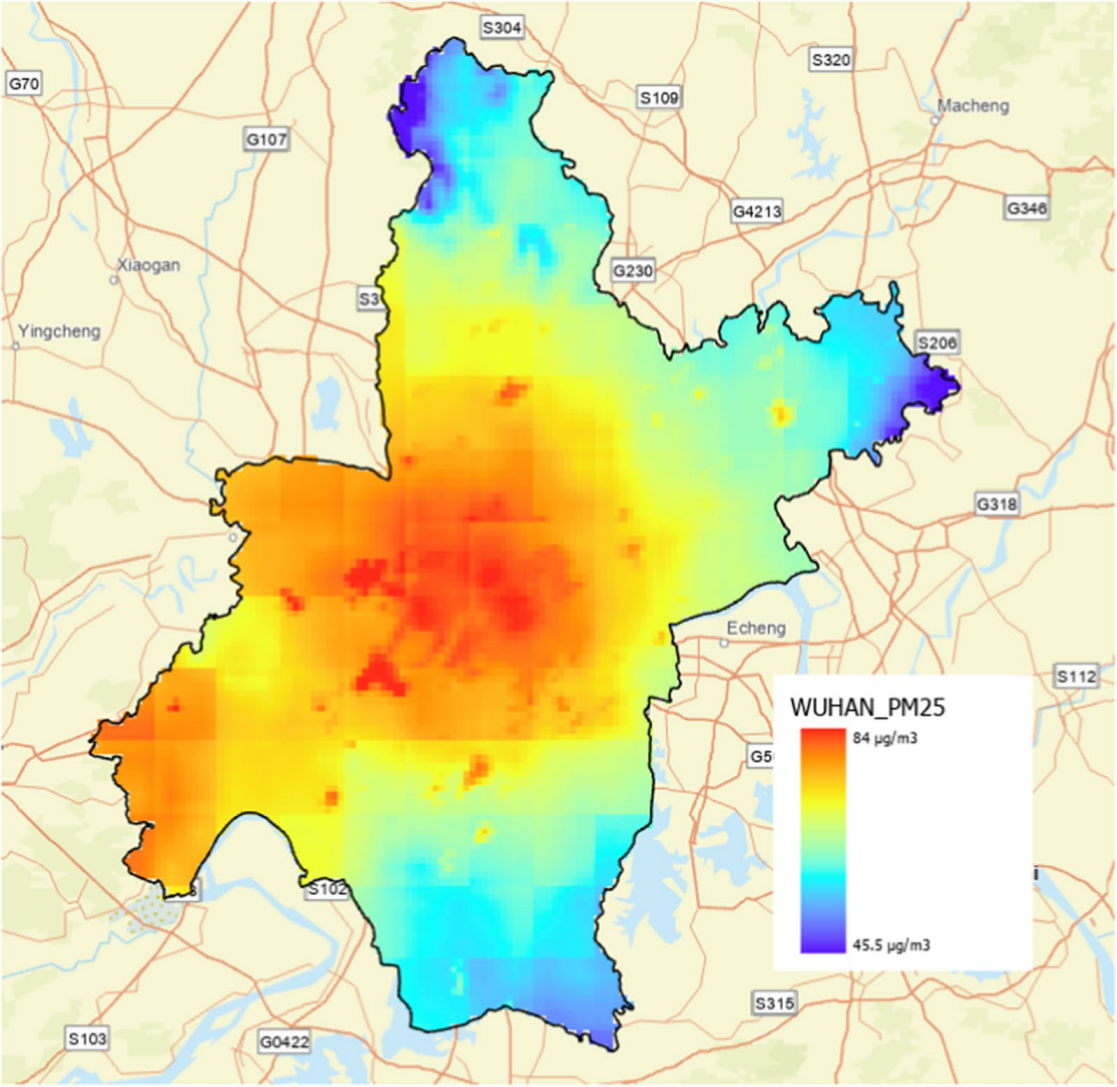
Annual PM_2.5_ concentration in Wuhan in 2014

An annual PM_2.5_ map in 2014 covering all districts of Wuhan was applied in this study to represent spatial variability of air pollution (Fig. 3), which was retrieved from the open dataset [12, 35]. This open dataset was an annual PM_2.5_ map (spatial resolution: 0.01° × 0.01°) derived by a land use regression (LUR) with satellite observations, chemical transport modelling, and ground-based monitoring. LUR is a common mapping technique to estimate the spatial distribution of air pollution based on multiple factors such as urban landscape, transportation network, and terrain [30, 34]. The results of Hammer et al. [12] were highly accurate (R^2^ = 0.81; slope = 0.90), thus, this open dataset could represent the micro-scale spatial variability of PM_2.5_ in Wuhan.

Hourly PM_2.5_ data retrieved from nine monitoring stations (Table 1) covering the study period (May 13–May 31, 2014), were applied to measure the temporal variability of ambient exposure. The following method based on the spatial varying data from the annual PM_2.5_ map and temporal varying data from monitoring stations was proposed to calculate the spatiotemporal concentration of PM_2.5_ at a certain location *p*_*i,j*_ in Wuhan at different times. This method was adopted from a previous study to measure spatiotemporal exposure to PM_2.5_ based on satellite-derived data, air quality monitoring stations, and GPS-based wearable devices [43]. Particularly, PM_2.5_ concentration ${C}_{i,j}^{t}$ at location *p*_*i,j*_ and time *t* can be calculated with the following formula:

1 $$C_{i,j}^{t} = C_{{{\text{base}}\;{\text{site}}}}^{t} + (C_{i,j} - C_{{{\text{base}}\;{\text{site}}}} ),$$

where *C*^*t*^_*base site*_ is the temporal varying PM_2.5_ concentration retrieved from the representative station at time *t*, *C*_*i,j*_ and *C*_*base site*_ is the spatial varying PM_2.5_ concentrations at location *p*_*i,j*_ and representative station retrieved from the annual PM_2.5_ map. The representative station to calculate PM_2.5_ concentration *C*^*t*^_*i,j*_ at location *p*_*i,j*_ and time *t* should meet the following requirements: (1) no obvious outliers during the study period; (2) a minimum difference between the real-time concentrations from the monitoring station and the concentration of the corresponding position in the annual PM_2.5_ map. This Eq. (1) assumes that the spatial distribution of ambient PM_2.5_ exposure varied depending on multiple factors (e.g., built environment, urban structure), and the overall concentration is affected by temporal varying factors (e.g., weather condition of a day) and estimates the spatiotemporal variability of PM_2.5_ exposure in high resolution across the study area.

**Table 1:** PM_2.5_ stations in the monitoring network in Wuhan

Monitoring station	Latitude	Longitude	
Donghu Liyuan	30.578°N	114.384°E	
Donghu Gaoxin	30.523°N	114.410°E	
Wujia Mountain	30.637°N	114.142°E	
Wuchang Ziyang	30.536°N	114.315°E	
Hankou Jiangtan	30.597°N	114.313°E	
Hankou Huaqiao	30.622°N	114.294°E	
Hanyang Yuehu	30.591°N	114.264°E	
Chenhu Qihao	30.302°N	113.863°E	
Qingshan Ganghua	30.628°N	114.383°E	

Blue triangles represent the air quality monitoring stations

### Dynamic population exposure to PM_2.5_ among taxi drivers

The daily trajectories for each taxi *i* were combined *TRIPS*_*i*_ = {*trip*_*i*_^*1*^, *trip*_*i*_^*2*^, *trip*_*i*_^*3*^,…} to measure the dynamic population exposure to PM_2.5_ among taxi drivers. As depicted in “GPS-based taxi trajectory data”, all trips along the road network were generated after data preprocessing and each trip consisted of the following attributes, the start and end time (*time*_*start*_ and *time*_*end*_), trip distance, and the turning point coordinates. The dynamic population exposure to PM_2.5_ among taxi drivers was estimated at the trip level, which can be expressed as:

2 $${\text{EI}}\left({\text{trip}}_{i}^{n}\right) = \lambda \int_{{\text{time}}_{\text{start}}}^{{\text{time}}_{\text{end}}} {\text{Ce}}(t)dt,$$

where EI(*trip*_*i*_^*n*^) is the population exposure level of taxi driver *i* during a trip *trip*_*i*_^*n*^, which is the integral of PM_2.5_ exposure where the driver was located with respect to time, λ is the air pollutant filtration coefficient considering a difference in PM_2.5_ exposure between the indoor environment of the taxi and the external environment. However, it was impossible to consider the internal and external protective measures (e.g., opening and closing windows, whether the driver wears a mask, whether there is air filtration equipment in the taxi). Given that this study assumed that all taxi drivers had similar air pollution filtering conditions in the working environment, λ was a fixed coefficient. Specifically, this study was based on a scenario in May 2014 when taxi drivers often opened windows when driving if the weather conditions were good, thus, this calculation was based on an open window scenario. Ce(t) is PM_2.5_ exposure in the environment where the taxi was located at time *t*. Based on the spatiotemporal data of PM_2.5_ concentration at the grid level estimated according to Eq. (1), the integral formula is transformed as follows:

3 $${\text{EI}}\left( {{\text{trip}}_{i}^{n} } \right) = \lambda \int_{{\text{time}}_{\text{start}}}^{{\text{time}}_{\text{end}} } {\text{Ce}}(t)dt = \lambda \sum\limits_{\text{grid}} {\left( {C_{\text{grid}}^{t} \times \Delta t} \right)},$$

where *C*^*t*^_*grid*_ is PM_2.5_ exposure of the grid at time *t*, *Δt* is the elapsed time of taxi in the corresponding grid and each trip is split according to its relationships with grids to calculate the summation of PM_2.5_ exposure in each segment.

Subgroup analysis was conducted to evaluate the overall effect throughout the study period and weekday/weekend effects. Hotspot analysis was implemented to visualize and analyses the spatial–temporal variability of dynamic PM_2.5_ exposure among the top 1% of taxi drivers who experienced higher and lower levels of air pollution for each subgroup (e.g., weekdays, and weekends). Specifically, we chose the top 1% of taxi drivers with high and low PM_2.5_ exposure and analyzed the spatiotemporal variations of their activities, respectively. To guarantee the validity and robustness of the results, only taxi drivers who operated at least ten weekdays or four weekends were analyzed.

Kernel density estimation (KDE) was applied to estimate the hotspots for the above-mentioned two groups of taxi drivers based on their origins and destinations on weekdays and weekends. KDE is a method for smoothing data that converts a set of recorded observations, presented as geographically referenced point data, into a continuous surface [17]. This surface reflects the intensity of individual observations across space and is commonly used for hotspot analysis of air pollution exposure (e.g., [15]). In this study, ArcGIS 10.5 software was used to implement the hotspot analysis with a cell size of 0.001° × 0.001°.

## Results

### Spatial and temporal variability of ambient PM_2.5_ exposure

According to the current "Air Quality Standards" adopted in China, a PM_2.5_ concentration below 35 μg/m^3^ is “good” air quality, a concentration ranging from 35 μg/m^3^ to 75 μg/m^3^ is “moderate”, a concentration ranging from 75 μg/m^3^ to 115 μg/m^3^ is “unhealthy for sensitive groups” and a concentration exceeding 115 μg/m^3^ is “poor” [25]. In addition, WHO’s air quality guideline recommends that the 24-h average PM_2.5_ exposure should not exceed 25 µg/m^3^ [40].

**Fig. 4:**
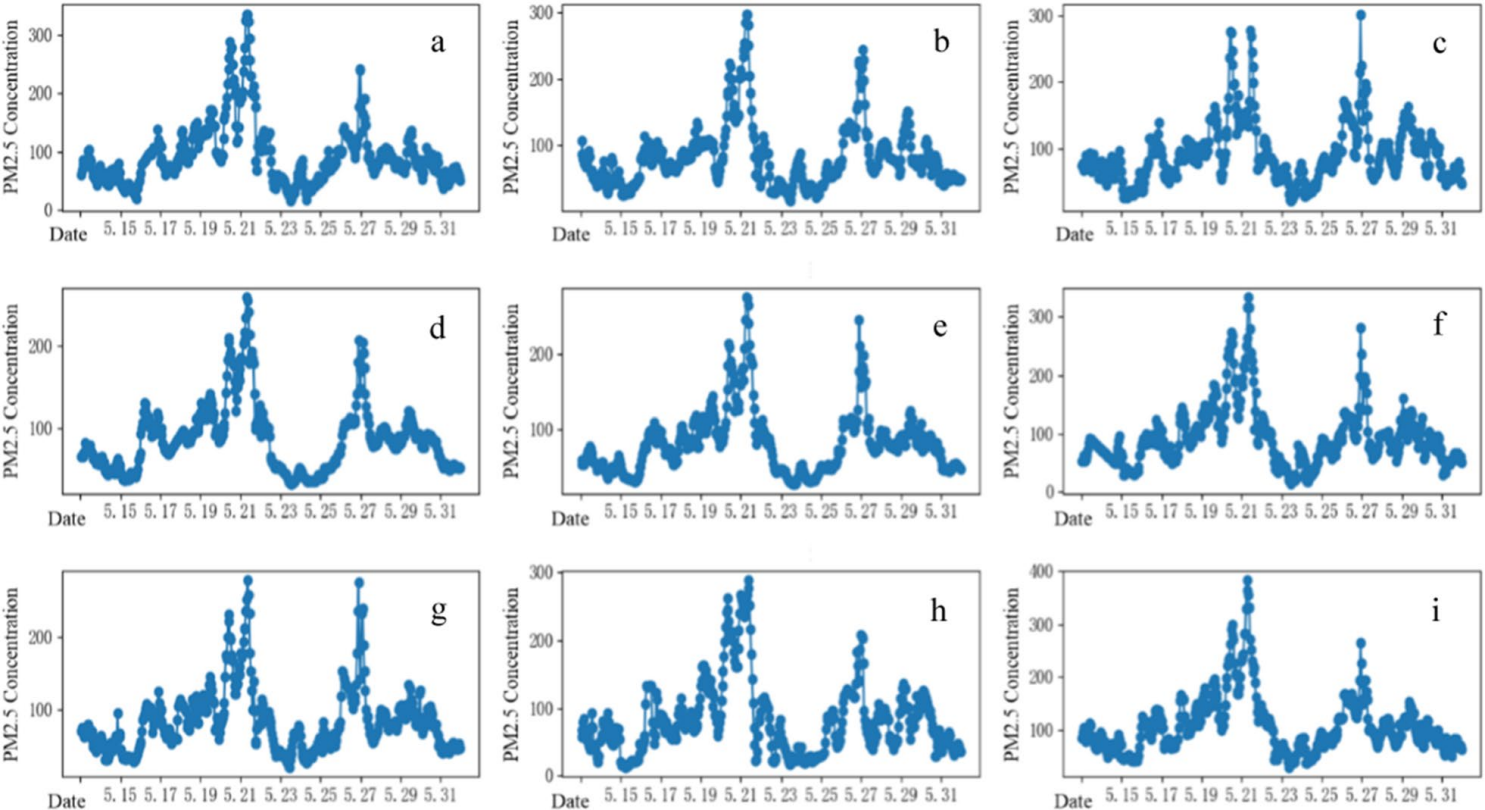
PM_2.5_ data collected from the monitoring stations in Wuhan. **a** Donghu Liyuan, **b** Donghu Gaoxin, **c** Wujia Mountain, **d** Wuchang Ziyang, **e** Hankou Jiangtan, **f** Hankou Huaqiao, **g** Hanyang Yuehu, **h** Chenhu Qihao, and **i** Qingshan Ganghua

Based on the spatial varying information retrieved from the annual PM_2.5_ map, the average PM_2.5_ concentration in Wuhan was 45.5 μg/m^3^, indicating an overall moderate air quality in Wuhan with higher pollution level in some central areas such as Qiaokou, Jianghan, Jiang'an, Wuchang, and Qingshan districts (Fig. 3). There was also high ambient PM_2.5_ exposure in the southwest of the Caidian and Hannan districts. However, a comparison of the temporal varying information retrieved from nine air pollution monitoring stations in Wuhan (Fig. 4) indicated that 13 days of the study period (May 13–May 31, 2014) had a pollution level above the threshold of “unhealthy for sensitive groups” (> 75 μg/m^3^), including 9 days with an ambient PM_2.5_ level exceeding 115 μg/m^3^. Furthermore, several days had pollution extremes. Ambient PM_2.5_ levels were almost 300 μg/m^3^. The peak PM_2.5_ pollution was between May 21, 2014 and May 27, 2014. In contrast, there were no significant regional differences between the nine monitoring stations in Wuhan, with a relatively consistent pattern of change over time observed. These results indicate that neither spatially varying data from the annual PM_2.5_ map nor temporal varying data from monitoring stations could comprehensively represent the spatiotemporal variability of PM_2.5_ exposure in line with our assumption to combine both datasets to better estimate population exposure.

### Hourly dynamic exposure of PM_2.5_ among taxi drivers

**Fig. 5:**
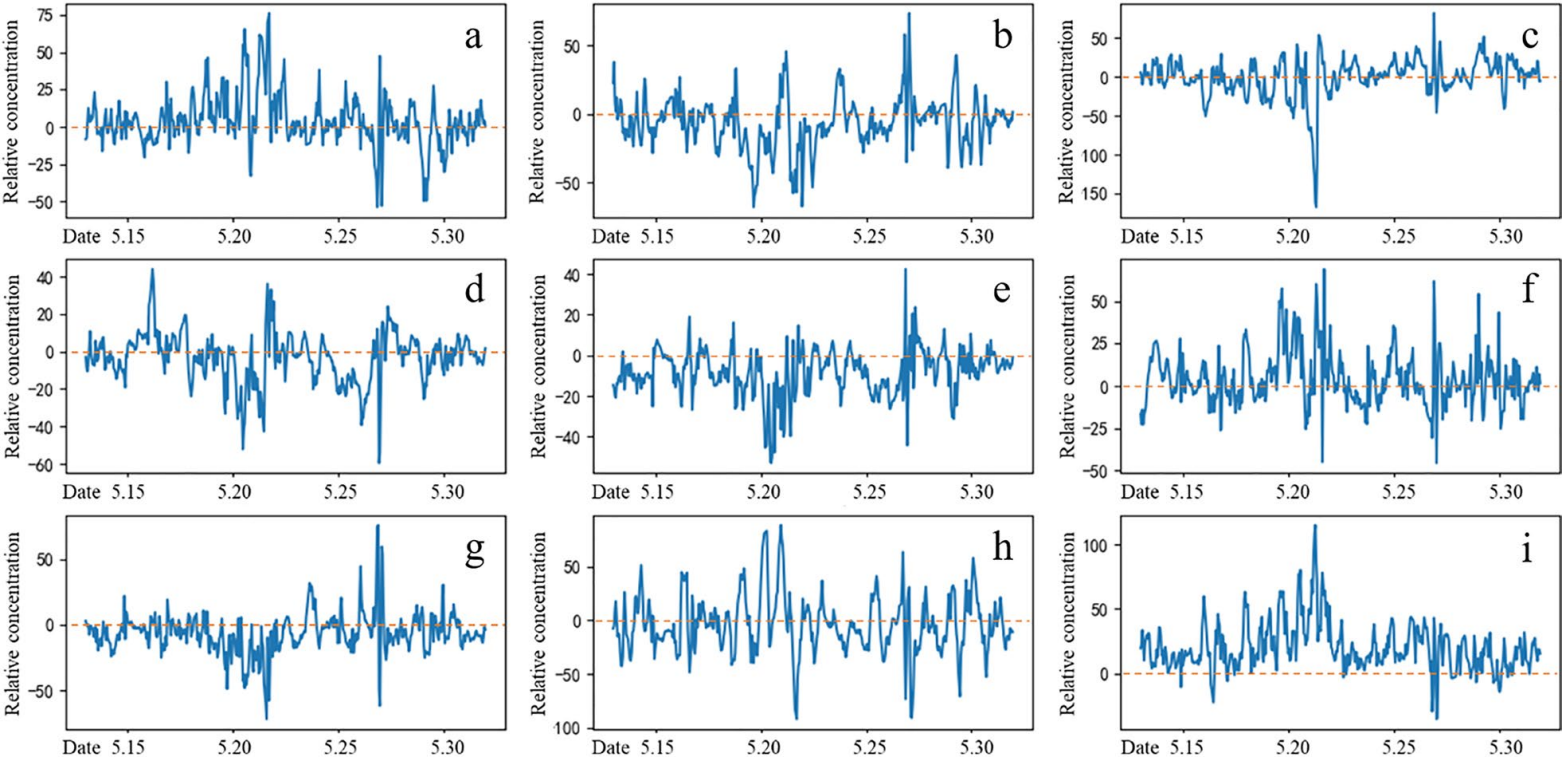
Concentration difference for each station in Wuhan. **a** Donghu Liyuan, **b** Donghu Gaoxin, **c** Wujia Mountain, **d** Wuchang Ziyang, **e** Hankou Jiangtan, **f** Hankou Huaqiao, **g** Hanyang Yuehu, **h** Chenhu Qihao, and **i** Qingshan Ganghua

To identify a representative station for estimating spatiotemporal variability of PM_2.5_ exposure, the differences in the data from all monitoring stations and the annual PM_2.5_ map, and the corresponding variances were calculated, as shown in Fig. 5 and Table 2. There was a large difference between May 21 and May 27, which was associated with severe pollution in Wuhan. The data distribution of all monitoring stations in the remaining days of the study period conformed to the annual distribution of PM_2.5_. The overall variances retrieved from Wuchang Ziyang (183.68 μg/m^3^) and Hankou Jiangtan (131.97 μg/m^3^) stations were the lowest, indicating better fitting as the representative monitoring station, thus Wuchang Ziyang station was selected as the representative station.

**Table 2:** The related variance for each station (μg/m^3^)

Station	a	b	c	d	e	f	g	h	i
Variance	308.04	355.41	657.53	183.68	131.97	253.47	238.32	747.47	358.51

**Fig. 6:**
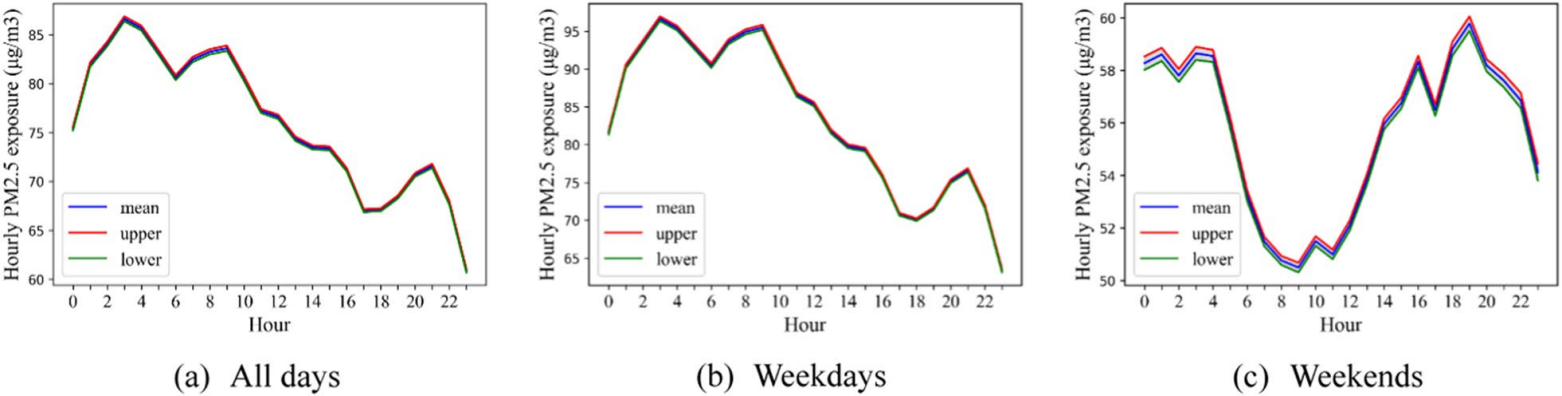
Hourly dynamics of PM_2.5_ exposure among taxi drivers in all days, weekdays and weekends across Wuhan during the study period. Mean represents the mean of exposure, upper and lower represent the 95% confidence interval of the exposure

Dynamic population PM_2.5_ exposure among taxi drivers at the trip level was then estimated (Fig. 6a), showing that there was higher PM_2.5_ exposure at midnight and in the morning (3:00–4:00 and 9:00–10:00 am). During these peaks, taxi drivers could be exposed to 86.61 μg/m^3^ (CI: 86.36–86.86 μg/m^3^) and 83.60 μg/m^3^ (CI 83.33–83.88 μg/m^3^) within an hour. These pollution levels were ~ 15.48% and 11.47% higher than the threshold for “unhealthy for sensitive groups”. However, the lowest average (11:00–12:00 pm) was only 60.90 μg/m^3^ (CI 60.71–61.10 μg/m^3^).

Additionally, PM_2.5_ exposure among taxi drivers during weekdays was generally higher than exposure at weekends (Fig. 6b, c). The peaks of weekday PM_2.5_ exposure were at midnight and morning with drivers exposed to above 95 μg/m^3^ within an hour between 3 and 9 am. In comparison, the peak hour of weekday PM_2.5_ exposure among drivers was in the evening and midnight and was ~ 59.79 μg/m^3^ (CI 59.51–60.06 μg/m^3^) at 7 pm during the weekend.

**Fig. 7:**
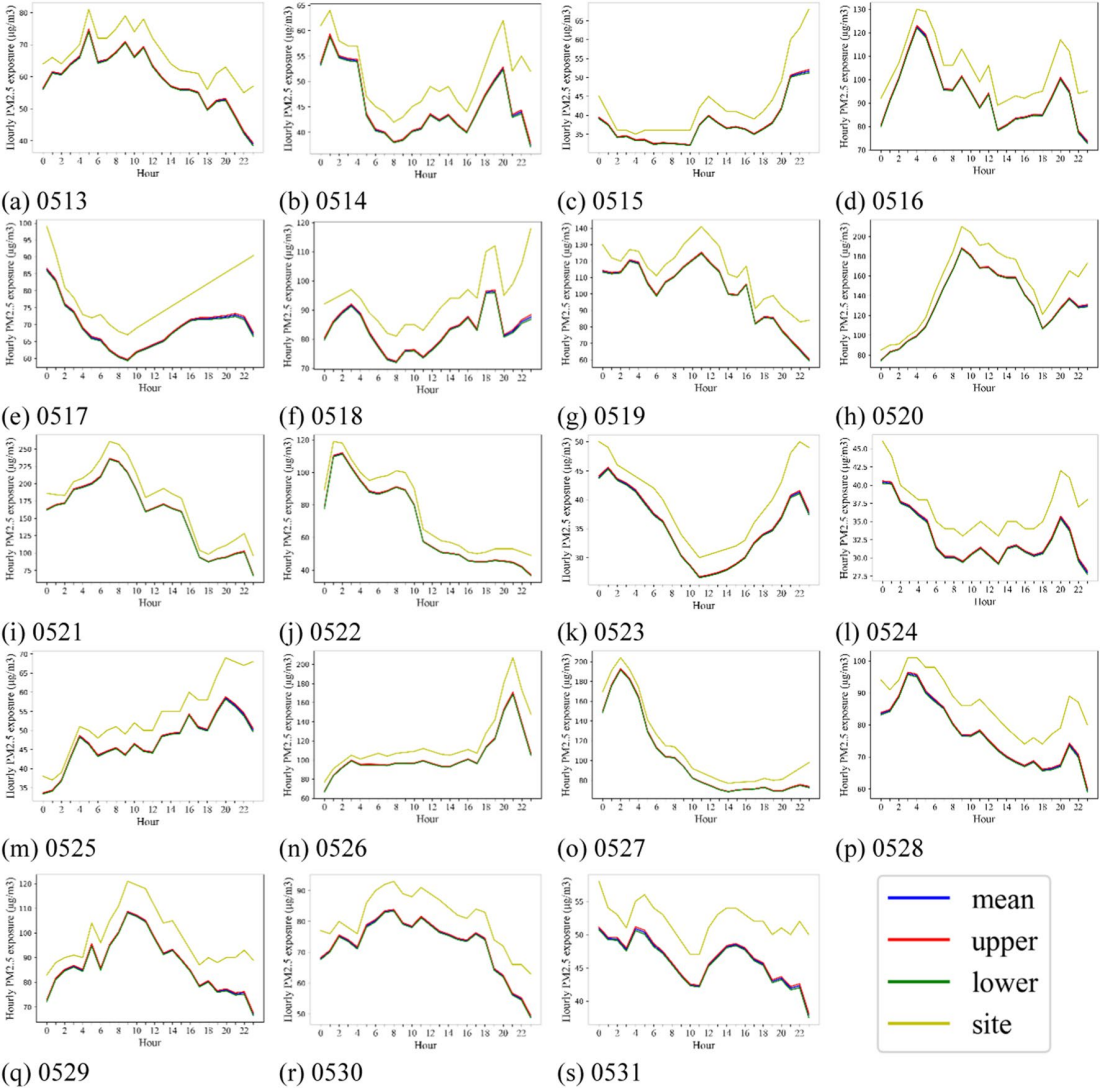
Hourly dynamic of PM_2.5_ exposure in each day between May 13–May 31. Mean represents the mean of exposure, upper and lower represent the 95% confidence interval of the exposure, site means the exposure based on the monitoring stations

The hourly dynamic of PM_2.5_ exposure was also highly varied by day (Fig. 7). Overall, the representative monitoring station overestimated the dynamic PM_2.5_ exposures of taxi drivers. For the days with higher pollution levels retrieved from monitoring stations, hourly variations of PM_2.5_ exposure among taxi drivers were larger than the other days. Specifically, the most polluted days (May 21 and May 27) had large variations, with averages of 156.45 μg/m^3^ and 101.14 μg/m^3^ and standard deviations of 11.79 μg/m^3^ and 7.99 μg/m^3^.

**Fig. 8:**
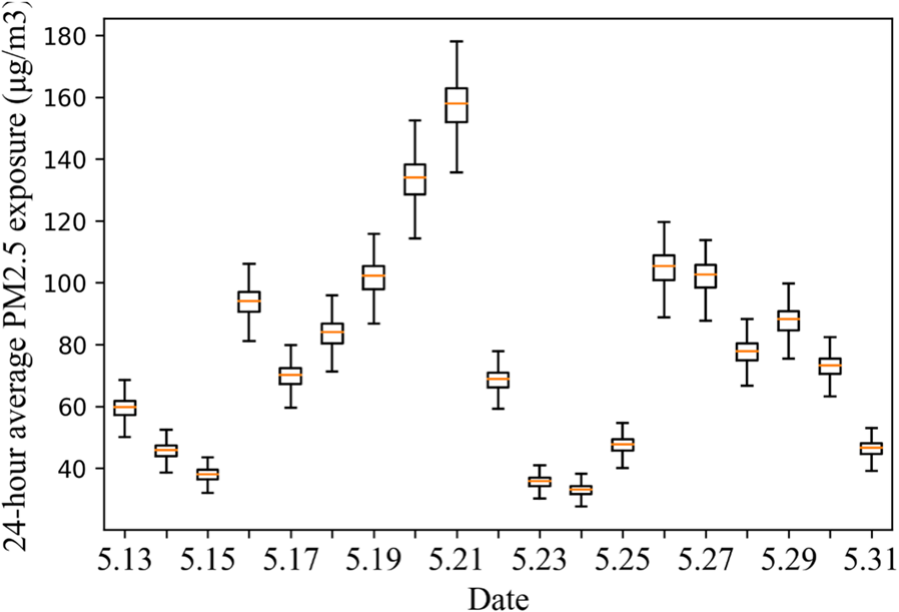
The daily 24-h average PM_2.5_ exposure among taxi drivers

Moreover, the 24-h average PM_2.5_ exposure among taxi drivers was calculated for each day (Fig. 8). During the weekdays, average 24-h PM_2.5_ exposure among taxi drivers was 83.60 μg/m^3^ (SD: 6.65 μg/m^3^), which was 234.4% higher than the WHO recommendation and 11.5% higher than the cutoff for “unhealthy for sensitive groups” noted in China’s guideline. During the weekend, 24-h average PM_2.5_ exposure among taxi drivers was 55.62 μg/m^3^ (SD: 4.72 μg/m^3^), which was approximately 25.8% lower than the pollution level during the weekdays. Although this pollution level did not reach the cutoff for “unhealthy for sensitive groups” noted in China’s guideline, it was still 2.2 times the WHO recommendation.

### Spatio-temporal analysis of taxi drivers’ activities

**Fig. 9:**
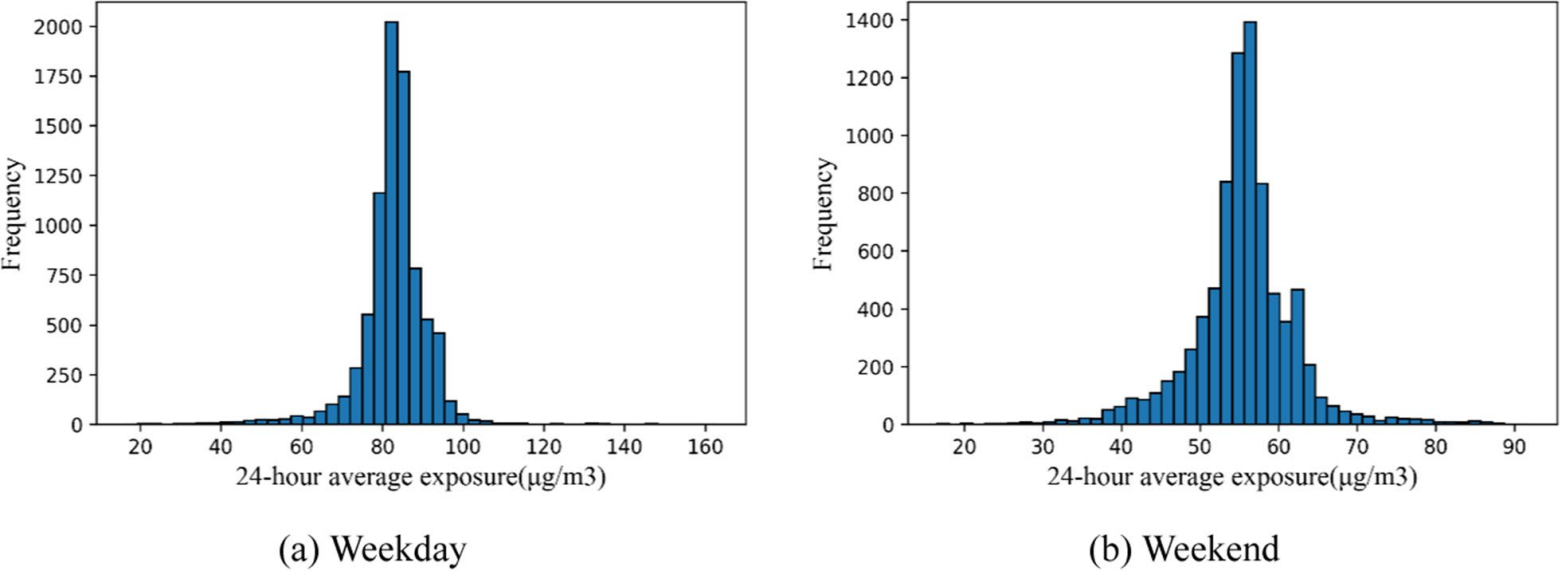
Distribution of the 24-h average PM_2.5_ exposure on weekdays and weekends across Wuhan in the study period

Before the comparison of the spatiotemporal variations of activities between taxi drivers with high and low PM_2.5_ exposure, the distribution of the 24-h average PM_2.5_ exposure on weekdays and weekends was explored (Fig. 9), showing a normal distribution on both weekdays and weekends. On weekdays, the 24-h average PM_2.5_ exposure ranged from 16.44 μg/m^3^ to 162.74 μg/m^3^, whereas the 24-h average PM_2.5_ exposure ranged from 16.57 μg/m^3^ to 91.70 μg/m^3^ on weekends.

**Fig. 10:**
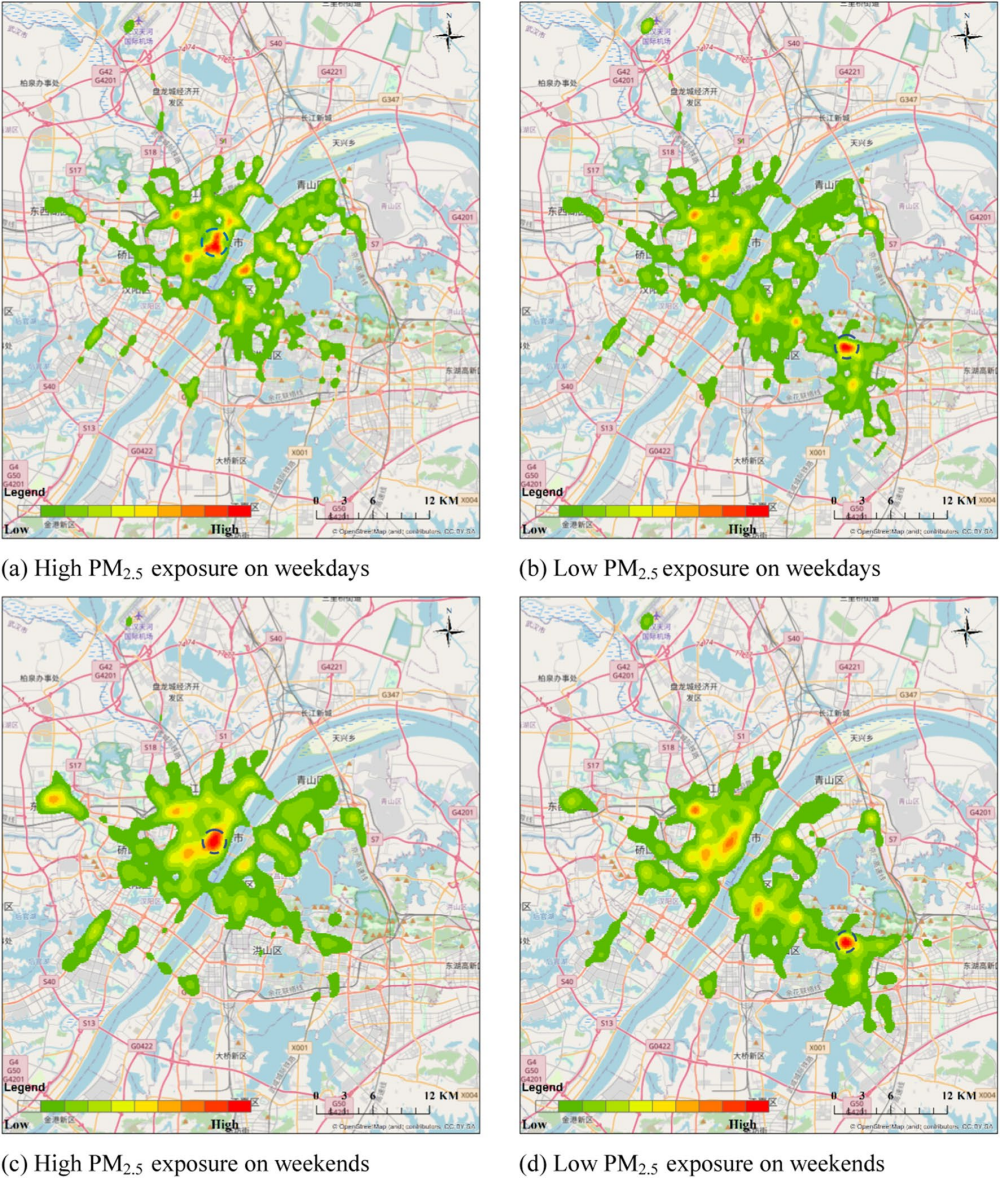
Hotspots of taxi drivers with high and low PM_2.5_ exposure on weekdays and weekends. As shown in **a**–**d**, where the dark red depicts the high-density areas, the high-density areas could correspond to the pollution hotspots

We further explored the difference among taxi drivers with different levels of PM_2.5_ exposure based on their travel patterns using KDE analysis. As shown in Fig. 10, the two groups presented a similar distribution of hotspots on weekdays and weekends respectively, while the patterns of the two groups differed on both weekdays and weekends. Specifically, taxi drivers with high PM_2.5_ exposure had smaller major activity areas but a longer average trip distance than those with low exposure. For taxi drivers with high PM_2.5_ exposure, the average trip distances on weekdays and weekends were 12.26 km and 12.12 km, respectively compared to 4.82 km and 4.84 km for the taxi drivers with low PM_2.5_ exposure.

The activity hotspots of the taxi drivers with high PM_2.5_ exposure were mainly in (1) busy riverside commercial areas within historic and central districts bounded by the “Inner Ring Road”, such as Jianghan Road (the marked red area in Fig. 10a, c) and Xudong, and (2) major transportation interchange/terminals (e.g., Hankou Railway Station). Specifically, Jianghan Road is a famous century-old commercial street in Wuhan located in the center of Hankou District with a total length of 1600 m. Jianghan Road and Xudong are also close to the Wuhan Yangtze River Tunnel and the Second Yangtze River Bridge, respectively, which are major roads with high traffic flow.

For the taxi drivers with low PM_2.5_ exposure, despite activity hotspots near major transportation interchange/terminals (e.g., Wuchang Railway Station), more hotspots overlapped with the new commercial areas in Wuhan, such as Optics Valley International Plaza (the marked red area in Fig. 10b, d) and Optical Valley Software Park within the “Third Ring Road”. These results suggested that the dynamic population exposure to PM_2.5_ among taxi drivers was related to their travel behavior.

## Discussion

### Interpretations of results

This study developed a method to rapidly assess dynamic population exposure among taxi drivers. An empirical study was conducted in Wuhan, China and subgroup analyses were performed to quantify population exposure in different scenarios (e.g., weekday/weekend effect). Our results indicated that neither spatially varying data from the annual PM_2.5_ map nor temporal varying data from monitoring stations could represent spatiotemporal variability of PM_2.5_ exposure independently. Based on the established equation to quantify dynamic population exposure among taxi drivers, it was found that taxi drivers are usually exposed to higher PM_2.5_ during the morning (3:00 am to 4:00 am). Furthermore, the peak hours of weekday PM_2.5_ exposure were at midnight and in the morning, while the peak hours of weekday PM_2.5_ exposure were during the evening and midnight. Additionally, taxi drivers with high PM_2.5_ exposure were typically clustered in commercial areas among central districts bounded by the “Inner Ring Road” and major transportation interchange/terminals, while taxi drivers with low PM_2.5_ exposure usually drove further in each trip with activity hotspots in new commercial areas bounded by the “Third Rind Road”, indicating a high spatiotemporal variability.

**Fig. 11:**
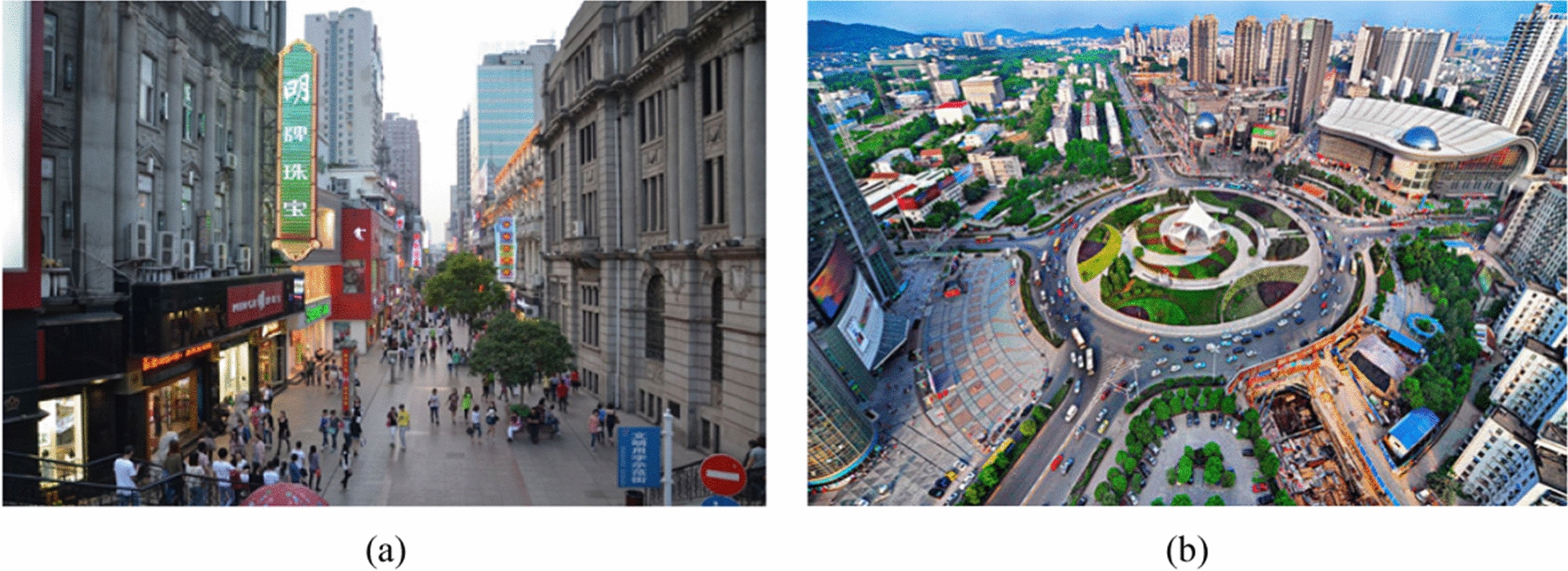
Two main hotspots: **a** Jianghan Road (Open-source photo from government site: http://www.jianghan.gov.cn/) and (**b**) Optics Valley International Plaza (Open-source photo from local newspaper: http://www.cnhubei.com/)

These results demonstrate how daily travel behaviors could affect the air pollution exposure of taxi drivers. For example, commercial areas in historic and central districts usually have high PM_2.5_ exposure due to the co-effects of road traffic and population mobility. These commercial areas are associated with PM_2.5_ pollution due to poor ventilation from high-rise, high-density urban morphology [28] and local emissions from buildings and electrics [22] with frequent road transit (e.g., bus, car) to/from commercial areas increasing the level of traffic-related PM_2.5_ exposure within a short period (e.g., in an hour). As a result, taxi drivers who frequently drive within commercial areas in central districts during high traffic volume and major commercial activities would be exposed to urban canyons with poor design and low ventilation that trap traffic- and building-related PM_2.5._ This hypothesis is supported by local evidence, for example, Jianghan Road (Fig. 11a) and Xudong are two major activity hotspots for taxi drivers with high exposure, Jianghan Road is a major commercial area in Wuhan with pedestrian zones covering facilities and landmarks for social, leisure, and tourist activities. According to the annual report on passenger flow in Wuhan (http://www.jianghan.gov.cn/mljh/zsyz/zsdt/201908/t20190816_3743.shtml), Jianghan Road had a high daily passenger flow (e.g., average passenger flow per day until 2019 = 400,000 people). Xudong is a central business district (CBD) in Wuhan with multiple hospitals, schools, and universities, as well as connections to various major roads (e.g., Heping Road, Youyi Road, the Second Yangtze River Bridge in Wuhan). These results may also explain the high PM_2.5_ exposure among taxi drivers in the morning during the weekday and in the evening during the weekend, i.e., peak working hours and peak times for social, leisure, and tourist activities, respectively.

In comparison, activity hotspots of taxi drivers with low exposure were in new commercial areas in extended urbanized areas such as Optics Valley International Plaza (Fig. 11b). Although these areas also have frequent road transit that can increase traffic-related PM_2.5_ exposure, they are better designed with better ventilation and a lower building density. Furthermore, these drivers need to drive further and pass through nearby districts for work which are usually residential areas or suburbs with more greenery and less traffic leading to lower PM_2.5_ exposure.

For major transportation interchanges/terminals, taxi drivers may be frequently exposed to PM_2.5_ pollution from both nearby road traffic and railway systems. Specifically, Hankou Railway Station and Wuchang Railway Station are two major train stations in Wuhan responsible for the departure or transit of northbound and southbound passengers every day. Thus, trains frequently pass through the railway systems near these stations daily. More importantly, there are several nearby bus terminals with a large flow of people and vehicles including interurban coaches and local buses as well as cars and taxis. This population dynamic potentially increases taxi drivers' exposure to traffic-related PM_2.5_ during the peak hour of transit and partially explains why taxi drivers could also be exposed to high PM_2.5_ exposure at midnight on both weekdays and weekends, as they might serve customs to connect to intracity trains and coaches.

The pollution episode between May 21—27 matched with the local news from China, for example, the People’s Daily reported that Wuhan had the second worst air pollution among 161 Chinese cities (http://env.people.com.cn/n/2014/0521/c1010-25044033.html), possibly due to regional climate but this requires further exploration.

### Health and policy implications

Based on the results, taxi drivers who work in urban areas could be at risk, especially those who frequently drive to commercial areas in central districts and major transportation interchanges. Specifically, the average PM_2.5_ exposure among taxi drivers during weekdays was already 11.5% higher than the WHO’s threshold and cutoff for “unhealthy for sensitive groups” noted in China’s guideline. Those who frequently drive to central districts and major transportation interchanges may be exposed to a higher level of PM_2.5_, thus taxi drivers in urban China could be at risk of severe health risks due to their high PM_2.5_ exposure during working hours.

It is important to note that long-term exposure to PM_2.5_ can induce lung cancer among the urban Chinese population. For example, a study of the Yangtze River Delta (YRD) region [36] found that ~ 12,574–14,504 lung cancer deaths were attributable to PM_2.5_ in YRD, and the deaths in urban areas could be 7–13 times higher than those in rural areas. Guo et al. [11] also found that long-term exposure to PM_2.5_ could result in a much higher incidence rate and mortality risk among urban males than rural males across 353 counties in China. Long-term exposure to PM_2.5_ in China is also associated with cardiovascular and respiratory diseases [20, 42]. Furthermore, frequently driving a taxi might also affect the driver’s physical activity and diet resulting in comorbidities such as obesity, hypertension, diabetes, and related chronic diseases that could further increase the risk of PM_2.5_ exposure.

Thus, it is important to provide community health training (e.g., program to enhance knowledge, attitude, and practice) to increase the health awareness of taxi drivers [24]. Taxi drivers should also enhance their environmental awareness, such as knowledge to install of air purifiers and attitude regarding frequently checking air filters. Additionally, knowledge to enhance healthy behaviors should also be provided to minimize potential risks from comorbidity. Raising such awareness among taxi drivers about the risks of PM_2.5_ exposure and providing guidance on protective measures can contribute to their well-being.

Additionally, strategies to improve urban design should also be applied to reduce the risk of long-term PM_2.5_ exposure. For example, urban greening should be applied along the major roads to remove air pollutants [18] and future major roads could be built along wind corridors to enhance urban ventilation so that less PM_2.5_ is trapped in the high-rise, high-density environment [7].

### Limitations

Several limitations need to be noted in this study for future research: (1) the representative station was near major traffic spots and although these stations were more statistically stable for mapping and analysis, the pollution level was much higher than the average dynamic exposure. Therefore, other types of monitoring stations should be used for comparison to quantify the local risk and differentiate different local scenarios (e.g., air pollution level in residential areas). (2) Our base map that represents spatial variability of PM_2.5_ exposure was from a nationwide model (spatial resolution: 1 km) and although this model is representative of the China scenario, enhanced results for local scale with finer resolution should also be applied to enhance the estimation. Specifically, it is recommended to use real-time traffic volume data and data from various emission sources for local modelling and validation in the future. (3) This study followed previous studies [43] to use the difference between the monitoring data and reference data for the temporal adjustment. However, for temporal adjusting spatially rich data, the ratio between the monitoring data and reference data in the background or the nearest monitoring site may also be appropriate, and different methods may yield the results. Future studies should compare the differences and uncertainties between various types of temporal adjustment. (4) As the data on taxi driver’s behaviors were retrieved from GPS information, we were not able to obtain sociodemographic information on each driver for further analysis, so future studies should consider using multiple sources of big data (e.g., taxi apps with drivers’ information) to enhance the data analysis.

## Conclusion

Understanding PM_2.5_ exposure is of significant importance due to its potential adverse effects on human health and the environment. Taxi drivers, due to their job nature, spend a significant amount of time on the road and may be exposed to varying levels of PM_2.5_. Concerns about population exposure to PM_2.5_ are particularly pertinent for taxi drivers, especially in urban and densely populated regions characterized by prevalent traffic-related pollution and other emission sources. Consequently, this study proposed a rapid assessment framework to estimate dynamic population exposure to PM_2.5_ among taxi drivers based on the annual PM_2.5_ concentration map, monitoring station data, and GPS-based taxi trajectory data. An empirical study in Wuhan, China was conducted to validate the proposed framework, showing that taxi drivers could be at risk of high PM_2.5_ exposure, especially those who frequently drove to major transportation terminals and within central districts. The research findings are beneficial for promoting air quality management and occupational risk prevention to mitigate potential health risks of taxi drivers.

## Data Availability

The datasets generated and/or analysed during the current study are not publicly available but may be available on reasonable request.
